# Estimation of ZIKV and DENV seroprevalence rates in Rio de Janeiro, Brazil, 2018

**DOI:** 10.11606/s1518-8787.2026060007181

**Published:** 2026-10-23

**Authors:** Geani de Oliveira Marins, Thiago de Oliveira Pires, Reinaldo Souza-Santos, Andréa Sobral, Rafael Freitas de Oliveira Franca, Elisa de Almeida Neves Azevedo, Marília de Albuquerque Sena, Raquel de Vasconcellos Carvalhaes de Oliveira, André Reynaldo Santos Périssé

**Affiliations:** I Fundação Oswaldo Cruz Escola Nacional de Saúde Pública Sérgio Arouca Programa de Pós-Graduação em Saúde Pública e Meio Ambiente Rio de Janeiro RJ Brasil Fundação Oswaldo Cruz. Escola Nacional de Saúde Pública Sérgio Arouca. Programa de Pós-Graduação em Saúde Pública e Meio Ambiente. Rio de Janeiro, RJ, Brasil; II Centro Universitário Adventista de São Paulo São Paulo SP Brasil Centro Universitário Adventista de São Paulo. São Paulo, SP, Brasil; III Fundação Oswaldo Cruz Escola Nacional de Saúde Pública Sérgio Arouca Departamento de Endemias Samuel Pessoa Rio de Janeiro RJ Brasil Fundação Oswaldo Cruz. Escola Nacional de Saúde Pública Sérgio Arouca. Departamento de Endemias Samuel Pessoa. Rio de Janeiro, RJ, Brasil; IV Fundação Oswaldo Cruz Instituto Aggeu Magalhães Recife PE Brasil Fundação Oswaldo Cruz. Instituto Aggeu Magalhães. Pernambuco, Recife, PE, Brasil; V Fundação Oswaldo Cruz Instituto Nacional de Infectologia Evandro Chagas Rio de Janeiro RJ Brasil Fundação Oswaldo Cruz. Instituto Nacional de Infectologia Evandro Chagas. Rio de Janeiro, RJ, Brasil

**Keywords:** Accuracy, Arbovirus Infections, Dengue, Zika, Rapid Diagnostic Tests

## Abstract

**OBJECTIVE:**

To estimate the accuracy of rapid tests for Zika and dengue in areas with cocirculation of flaviviruses and their prevalence rates in the municipality of Rio de Janeiro, Brazil, using methodologies for correction of partial verification bias.

**METHODS:**

A study was conducted based on a seroepidemiological survey. Participants underwent rapid tests for Zika and dengue. Venous blood samples were collected from participants with positive rapid test results for Zika and/or dengue, for diagnostic confirmation by the plaque reduction neutralization test (PRNT). To estimate prevalence, sensitivity and specificity parameters of the rapid tests were verified using the correction methodology proposed by Begg and Greenes (1983).

**RESULTS:**

A total of 2,120 participants underwent rapid test, and 1,087 (51.2%) were indicated for performing PRNT. The samples from 769 participants underwent PRNT, resulting in 686 (89.2%) positive samples for dengue and 522 (67.8%) for Zika. The sensitivity rates corrected for partial verification bias were 81.0% and 91.5% for the dengue and Zika rapid tests, respectively. The corrected specificity rates were 66.7% for the dengue rapid test and 77.1% for the Zika rapid test. The estimated population prevalence rates, corrected for rapid test accuracy, were 88.0% for dengue (95%CI: 86.7–89.4) and 41.6% for Zika (95%CI: 38.6–44.5).

**CONCLUSIONS:**

After correction for partial verification bias, the Zika rapid test performed better than the dengue rapid test in the scenario of arbovirus cocirculation. Our findings indicate that the population of the municipality of Rio de Janeiro is predominantly exposed to the dengue virus.

## INTRODUCTION

According to the Pan American Health Organization^1^, dengue is the most prevalent arbovirus disease in the Americas. In 2015 and 2016, Brazil faced a triple epidemic caused by the Zika, dengue, and Chikungunya arboviruses, and the municipality of Rio de Janeiro (MRJ) was seriously affected. The incidence rates in the MRJ in 2016 were 496 cases/10,000 inhabitants for Zika and 391.5 cases/10,000 inhabitants for dengue. In the same year, up to epidemiological week 49, 263.598 probable cases of Chikungunya were reported nationwide, corresponding to an incidence rate of 128.9 cases/100 thousand inhabitants. In 2016, Zika surpassed dengue in incidence, although dengue remained highly prevalent in MRJ, even after the introduction of the Zika virus (ZIKV) in 2015^2^.

Brazil is among the six countries with the highest dengue virus (DENV) incidence in the Americas so far in 2025, notwithstanding the decrease in ZIKV incidence compared to the same period in 2022^2^. In Rio, in 2024, dengue was declared a public health emergency by the mayor of the MRJ^3,4^ and in the first four epidemiological weeks of 2025, dengue incidence in the MRJ was 292 cases/100 thousand inhabitants^2^. No cases of Zika were reported, and 109 cases of Chikungunya were confirmed in RJ during the same period. Information on asymptomatic individuals is limited due to underreporting^5^.

In Brazil, the diagnostic test for detecting DENV and ZIKV is reverse transcriptase polymerase chain reaction (RT-PCR). However, the need to optimize laboratory diagnosis using cheaper and more accessible screening tests has led to the more widespread use of point-of-care immunochromatographic rapid tests (RTs)^6^. Nevertheless, studies indicate variability in the accuracy of DENV and ZIKV RTs, with sensitivity and specificity values less than 50%^7,8^.

RTs cannot distinguish between the different DENV serotypes, and cross-reactivity is possible between DENV and ZIKV antibodies and yellow fever vaccination. In this case, the plaque reduction neutralization test (PRNT) is considered the gold standard for detecting neutralizing antibodies to arbovirus infections and is the most specific serological test for flaviviruses, being the recommended test in endemic areas for DENV and ZIKV^9^. Still, the method is labor-intensive and requires highly trained personnel and the use of live virus^10^.

DENV and ZIKV infections make the scenario even more complex because they may be asymptomatic or subclinical. When they are symptomatic, they may be confused with other viral infections due to their nonspecific clinical presentations. According to epidemiological data, an estimated 80% of people infected with DENV and ZIKV are asymptomatic^11^. In addition, understanding the clinical variability is insufficient in regions such as Rio de Janeiro with hyperendemicity and coinfection^12^.

Serological surveys allow mapping cases not detected by epidemiological surveillance and elucidate the dynamics of circulation of arbovirus infections through random testing of individuals in a given geographic space^5^. However, these studies use complex sampling, and although they are advantageous and highly relevant, they may present the loss or absence of responses. Partial verification bias (PVB) occurs when the result of the index test, in our case the RT, interferes in the decision to apply the gold standard test (PRNT in our study) in only one group of participants^5^. Our study used the database from the ZDC Project (Zika, Dengue and Chikungunya seroprevalence in the MRJ), which was not initially intended to evaluate the accuracy of RT but rather to confirm the positive RT results. For this reason, only samples with positive results were subjected to the reference test, whereas negative samples were not retested. Thus, the exclusion of participants who may have been classified erroneously as negative by the index test may underestimate or overestimate the sensitivity and specificity.

We aimed to evaluate the accuracy of RT for DENV and ZIKV in regions with cocirculating flaviviruses, as well as to determine their prevalence rates in the MRJ, Brazil, while employing methodologies to correct for PVB.

## METHODS

### Study Design and Area

This study used the database from the ZDC Project^5^, a cross-sectional study conducted in the MRJ, the capital of the state of Rio de Janeiro, Brazil, from July 1 to October 31, 2018.

In 2018, MRJ had an estimated population of 6,688,927, with a total territory of 1,255.3 km^2^. The city has a hot, humid tropical climate with an annual temperature between 20 and 30ºC and abundant rainfall in the summer. The city is also characterized by numerous favelas, where people live in precarious socioeconomic conditions. The MRJ has the second largest gross domestic product in Brazil, but ranks 45^th^ in the human development index (0.799)^13^. MRJ has 33 administrative regions and 163 neighborhoods^14^.

### Selection of Participants

The study was based on a sample of volunteers recruited randomly from census tracts used by the *Instituto Brasileiro de Geografia e Estatística* (IBGE - Brazilian Institute of Geography and Statistics), according to the geographic operational base of the 2010 Population Census, ordered according to administrative regions^15^.

The sample included volunteers living in permanent private households in Rio de Janeiro. Eligible individuals received home visits to conduct the interviews and draw blood samples. The interviews were conducted in home visits carried out from Monday to Friday, between July 1 and October 31, 2018, by previously trained professionals.

Information was collected using a questionnaire based on a document produced by the World Health Organization (WHO), which aims to standardize studies on the prevalence of arbovirus infections worldwide^16^. The questionnaire collected sociodemographic information (sex, race/skin color, age, and marital status) as well as a history of yellow fever vaccination.

The ZIKV and DENV RTs were performed with fingerstick blood samples immediately after the interviews. As a strategy to reach individuals who had not been interviewed or had not undergone RTs during the week, a second visit was scheduled on weekends (Saturdays and Sundays). The RT results were available within 15 to 20 minutes. RT-positive individuals for ZIKV and/or DENV (except under-five children) had venous blood samples drawn for subsequent PRNT during the study period.

In this study, positive samples for DENV and/or ZIKV according to the rapid test (IgM/IgG RT) and confirmed by PRNT were defined as cases of infection.

### Laboratory Procedures

The RT kits used here were based on the immunochromatographic Dual-Path Platform (DPP®), with simultaneous detection of IgM and IgG for the target arboviruses. The kits were produced by Biomanguinhos (*Fundação Oswaldo Cruz*) in partnership with Chembio Diagnostic Systems, Inc. The reading was performed after 15 to 20 minutes. The results are interpreted according to the reading of the absolute numbers for the established cutoff points, namely: ZIKV IgM/IgG – nonreactive 0 to 22, indeterminate ≥ 23 to ≤ 27, and reactive ≥ 28; DENV IgM/IgG – nonreactive ≤ 18, indeterminate > 18 to < 22, and reactive ≥ 22.

The samples for performing PRNT were sent to the Laboratory of Virology and Experimental Therapy of the Aggeu Magalhães Institute, *Fundação Oswaldo Cruz* for diagnostic confirmation and detection of cross-reactivity between the flaviviruses. The biological material was transported in a cooler with a digital thermometer and reusable icepacks at 2ºC–8ºC and stored in a freezer (-70ºC).

PRNT was performed according to the standard protocol. Briefly, we used Vero strain cells (300,000 cells/mL) in a culture medium (minimal essential medium, GIBCO) with 10% FBS added in microplates with 24 wells (0.5 mL/well) prepared 48 hours before the assay. The serum samples were inactivated (30 minutes at 56ºC) and diluted (1/20, 1/80, 1/320, and1/1,280) in microplates with 96 wells and subsequently incubated with the virus (DENV1 BR-PE/97-42735, DENV2 BR-PE/95-3808, DENV3 BR-PE/02-95016, ZIKV PE243) at a concentration containing 30–70 PFU/mL. After incubation (37°C, 5% CO2) for one hour, the plates with 24 wells were inoculated in duplicate with 50μl of each virus/serum mixture and incubated (37°C, 5% CO2) for one hour. After incubation, the cells were covered with 500 μl of semisolid medium. After incubation for six days with the flaviviruses, the cells were fixed with 3.5M formalin, stained with crystal violet, and counted.

The neutralizing antibody titers were determined by a reduction of 50% or greater in the number of plates (PRNT50) at the lowest serum dilution (1:20) compared to the virus samples inoculated at the same dilution. The antibody titers were calculated with nonlinear regression and transformed into a log scale (log10).

Negative results for ZIKV and DENV were defined as the absence of detectable antibodies in serum samples, together with the presence of virus-induced cytopathic effects, followed by extensive plaque formation in cell cultures.

### Sample Size

We used a complex two-stage cluster sampling design. The primary units for selection in the study were the census tracts of MRJ, according to the 2010 Population Census (IBGE), ordered by administrative region and then by mean household income within the tract. The secondary units were households. Tracts were selected using probability proportional to size, with the number of private households in each tract, as per the 2010 Census, serving as the measure of size. The study’s sample size was calculated to provide sufficient statistical power to estimate the prevalence rates of 1.5% or higher for reported Zika and dengue cases. It was determined that the relative margin of error should be a maximum of dR = 35% (corresponding to an absolute error of 0.00525), with a 95% confidence coefficient (1 - α). The sample size was multiplied by an estimate of the sampling plan effect^17^, defined as 2.15. Thus, the sample size was calculated as 4,229. According to the 2010 Census, the mean number of residents per household was 2.93, so the sample size of households was 1,535. According to field logistics, it was determined that in each selected census tract, 10 households should be interviewed to estimate prevalence rates > 1.5%, resulting in a sample size of 152 tracts.

To correct for the non-response rate, sample weights were calibrated to known population totals by age and sex; to adjust for bias in the estimates, a sample weight was applied to each participant^5^.

### Analysis and Correction of Bias

Serological surveys use complex sampling and, although they are advantageous and highly relevant, they may show the loss or absence of responses^18^. PVB occurs when the result of the index test, in our case the RT, interferes with the decision to apply the gold standard test (PRNT in our study) in only one group of participants^19^.

For this purpose, we used the correction for calculating sensitivity and specificity proposed by Begg and Greenes^20^ and adapted by Alonzo^21^ for the diagnostic outcomes of dichotomous index tests, considering only RT-positive ZIKV and/or DENV cases. The authors used Bayes’ theorem to correct the bias, assuming that the mechanism for missing data is random, that is, the disease state (D) measured by the reference standard is considered randomly absent^21^.

Thus, with the bias correction, verification data missing according to the gold standard were estimated using the proportions of participants verified by PRNT to calculate the expected number of positive and negative participants among individuals not verified by PRNT. Substituting the empirical estimates, the sensitivity corrected for verification bias can be rewritten as follows: (1), D = 1, presence of the disease; D = 0, absence of the disease; T = status of the index test (positive = 1; negative = 0); s_1_ = positive for both tests; s_0_ = negative for the index test and positive for the reference standard; r_1_ = positive for the index test and negative for the reference standard; r_0_ = negative for both tests; u_1_= verification of the disease not obtained in positive individuals; u_0_ = verification of the disease not obtained in negative individuals; n _1_ = (s_1_+ r_1_+ u_1_); n_0 =_ (s_0_ + r_0_ + u_0_)_._:

$$\text{ Sensitivity ( }\alpha \text{ ): }\;\hat{P}(T=1|D=1)=\frac{\frac{s_{1}n_{1}}{s_{1}+r_{1}}}{\frac{s_{1}n_{1}}{s_{1}+r_{1}}+\frac{s_{0}n_{0}}{s_{0}+r_{0}}}$$

(1)

Likewise, corrected specificity can be calculated using the following formula (2):

$$\text{ Specificity ( }\beta \text{ ): }\;\hat{P}(T=0|D=0)=\frac{\frac{r_{0}n_{0}}{s_{0}+r_{0}}}{\frac{r_{0}n_{0}}{s_{0}+r_{0}}+\frac{r_{1}n_{1}}{s_{1}+r_{1}}}$$

(2)

We also assessed the positive likelihood ratio (PLR) and negative likelihood ratio (NLR). These calculations were performed using the results of the corrected accuracy parameters of the RT, as follows (3):

$$\mathrm{PLR}=\frac{\text{ sensitivity }}{1-\text{ specificity }}\;$$

(3)

$$\;\mathrm{NLR}=\frac{1-\text{ sensitivity }}{\text{ specificity }}$$

(3)

To correct the differential nonresponse bias, weighting of sampling weights was applied using an imputation based on demographic characteristics, such as sex and age data. To calculate the corrected ZIKV and DENV prevalence, we used the calculation proposed by Rogan and Gladen^22^. With *t*ˆ as the estimated frequency of positive tests, α as the corrected sensitivity, and β as the specificity, the corrected prevalence was defined as follows:

$$\hat{p}=\frac{\hat{t}+\alpha -1}{\alpha +\beta -2}$$

(4) 

The 95% confidence intervals were calculated for the prevalence estimates. All analyses were performed using the R software, version 4.3.1. The sampling plan and weight were incorporated into the point and interval estimates using the survey package^23^.

### Ethical Aspects

The “ZDC Project” was approved by the Institutional Review Board of the Escola Nacional de Saúde Pública Sérgio Arouca, under review number CAE 83186318.1.0000.5240.

## RESULTS

A total of 4,386 potential volunteers were selected for the study. Volunteers were included from 914 households, that is, 2,120 individuals underwent serological RTs. Of these, 51.2% were indicated for PRNT due to positive RT results for ZIKV and/or DENV. After eliminating individuals that declined to undergo venipuncture, PRNT was performed on samples from 769 volunteers, accounting for 70.7% of those indicated for the latter test (Figure).

**Figure:**
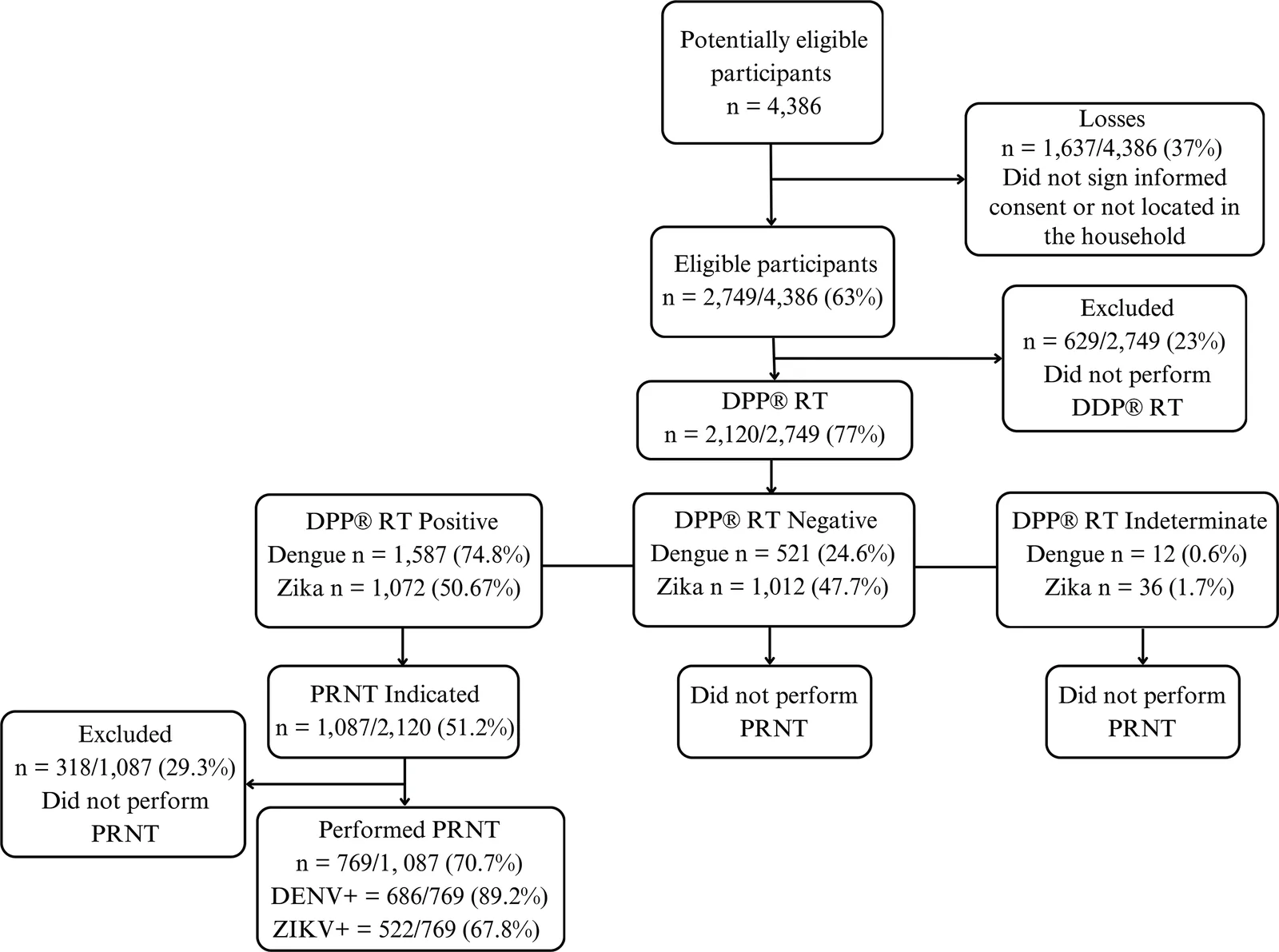
Flowchart of participants’ selection for the study.

RT: rapid test; PRNT: plaque reduction neutralization test; DPP: Dual-Path Platform;DENV+: positive for DENV. Note: DENV1 - 308 (45.0%), DENV2 - 210 (30.6%), and DENV3 - 650 (94.7%); ZIKV+: positive for ZIKV.

The predominant serotype was DENV-3 (94.7%), and 492 samples submitted to PRNT were positive for both DENV and ZIKV. The population estimate of individuals with positive PRNT results for both viruses was 23.6% (n = 1,578,348).

Among the participants, there was a predominance of females (53.1%), Blacks (65.2%), and individuals 30–59 years of age. More than half of the sample (55.9%) had been vaccinated for yellow fever. These results can be consulted in the previous study^5^.

When calculating corrected sensitivity and specificity, indeterminate RT results for ZIKV (n = 36) and DENV (n = 12) were removed from the analysis, resulting in 2,108 observations for DENV and 2,084 for ZIKV. Study participants whose blood samples (obtained from fingerstick) tested negative for ZIKV and/or DENV or yielded indeterminate results in the RT were not contacted for a subsequent venous blood collection and PRNT testing. This occurred because the ZDC Project was initially focused on the laboratory confirmation of cases with positive RT results, given the potential cross-reactivity between ZIKV and DENV. The corrected RT sensitivity and specificity values were 81.0% sensitivity and 66.7% specificity for the DENV RT and 91.5% sensitivity and 77.1% specificity for the ZIKV RT. The likelihood of having the disease increased fourfold given the ZIKV RT result (Table 1).

**Table 1:** Corrected validity parameters for rapid tests (IgG) for DENV (n = 2,108) and ZIKV (n = 2,084) in relation to the plaque reduction neutralization test (n = 769) in the municipality of Rio de Janeiro, Brazil, 2018.

Dengue						
	PRNT positive (s)	PRNT negative (r)	Verification not obtained (u)	Total (n)	Corrected sensitivity	Corrected specificity
RT positive (T = 1)	679 (s_1_)	27 (r_1_)	881 (u_1_)	1,587 (n_1_)	81.0^a^	66.7^a^
RT negative (T = 0)	7 (s_0_)	2 (r_0_)	512(u_0_)	521(n_0_)		
**Zika**						
	**PRNT positive (s)**	**PRNT negative (r)**	**Verification not obtained (u)**	**Total (n)**	**Corrected sensitivity**	**Corrected specificity**
RT positive (T = 1)	519 (s_1_)	186 (r_1_)	367 (u_1_)	1,072 (n_1_)	91.5^a^	77.1^a^
RT negative (T = 0)	3 (s_0_)	21 (r_0_)	988 (u_0_)	1,012 (n_0_)		

RT: rapid test; PRNT: plaque reduction neutralization test; S_1_: positive for both tests; s_0_: negative for RT and positive for PRNT; r_1_: RT positive and negative for PRNT; r_0_: negative for both tests; n_1:_ total RT positive; n_0:_ total RT negative; u_1_: verification of disease not obtained in positives; u_0:_ verification of disease not obtained in negatives; correction of accuracy (24).

^a^ Estimate considering the sampling weight. Dengue: uncorrected sensitivity: 98.3^a^; uncorrected specificity: 34.5^a^; Zika: uncorrected sensitivity: 99.6^a^; uncorrected specificity: 64.6^a^.

DENV and ZIKV crude (no corrections applied) prevalence rates were 32.4% and 25.4%, respectively (Table 2). The corrected population prevalence estimates, based on RT accuracy, were 88.0% for DENV and 41.6% for ZIKV. The two arbovirus infections were more prevalent in males and black people. Corrected dengue population prevalence increased gradually with age and was most prevalent in persons aged 30–59 years, while corrected Zika seroprevalence was highest in the age group over 19 years. Corrected population prevalence was higher in individuals without yellow fever vaccination (Table 2).

**Table 2:** Corrected population prevalence estimates for dengue and Zika in relation to sociodemographic characteristics of the study population, municipality of Rio de Janeiro, Brazil, 2018.

Variable	Category	Estimated population prevalence	
		Dengue	Zika
Total uncorrected estimate^a^	n^b^(Nˆ)^c^ % (95%CI)^c^	686 (2,168,023) 32.4% (28.8–36.1)	522 (1,702,034) 25.4 (22.1–29.0)
Total corrected estimate	n^b^(Nˆ)^c^ % (95%CI)^c^	1,587 (5,073,410) 88.0 (86.7–89.4)	1,072 (3,417,324) 41.6 (38.6–44.5)
Sex			
Female	n^b^(Nˆ)^c^ % (95%CI)^c^	934 (2,618,116) 86.0 (84.2–88.0)	625 (1,697,192) 39.5 (36.9–42.2)
Male	n^b^(Nˆ)^c^ % (95%CI)^c^	653 (2,455,295) 91.0 (88.9–92.8)	447 (1,720,132) 43.7 (40.3–47.0)
Race/color			
Black	n^b^(Nˆ)^c^ % (95%CI)^c^	921 (3,303,342) 91.9 (90.4–93.5)	654 (2,299,973) 45.5 (44.6–50.3)
Non–Black	n^b^(Nˆ)^c^ % (95%CI)^c^	666 (1,770,068) 83.0 (80.5– 85.4)	418 (1,117,351) 33.9 (30.9–37.0)
Age group (years)			
0 to 11	n^b^(Nˆ)^c^ % (95%CI)^c^	57 (445,316) 24.2 (16.8–31.7)	33 (257,393) 4.2 (0.7–7.7)
12 to 18	n^b^(Nˆ)^c^ % (95%CI)^c^	97 (541,046) 56.5 (48.9–64.1)	61(313,013) 21.8 (15.5–28.2)
19 to 29	n^b^ (Nˆ)^c^ % (95%CI)^c^	201 (997,120) 93.5 (90.5–96.2)	140 (729,461) 46.6 (40.5–52.8)
30 to 59	n^b^(Nˆ)^c^ % (95%CI) ^c^	765 (2,269,397) 97.3 (96.2–98.3)	512 (1,518,235) 45.3 (42.2–48.5)
≥ 60	n^b^(Nˆ) ^c^ % (95%CI) ^c^	467 (820,532) 92.8 (90.8–94.9)	326 (599,222) 46.9 (42.9–50.9)
Prior yellow fever vaccination			
Yes	n^b^(Nˆ)^b^ % (95%CI)^c^	868 (2,730,229) 84.3 (82.2–86.3)	566 (1,872,034) 37.6 (34.8–40.4)
No	n^b^(Nˆ)^c^ % (95%CI)^c^	696 (2,286,103) 93.6 (92.0–95.2)	490 (1,510,712) 47.0 (43.8–50.3)

Blacks: Blacks and Browns; Non-Black: Whites, Asian-descendants, Indigenous, and other; 95%CI: 95% confidence interval.

^a^ Estimate according to the result of the plaque reduction neutralization test without correction of accuracy and prevalence.

^b^ Sample.

^c^ Correction considering sampling weight and plan.

## DISCUSSION

We conducted a serological survey with complex sampling in Rio de Janeiro, an endemic city for DENV and ZIKV in 2018. In this complex epidemiological scenario, we assessed the diagnostic test considering partial verification bias to avoid serious overestimation of sensitivity and underestimation of specificity. Corrected sensitivity estimates for DENV (81.0%) and ZIKV (91.5%) were lower than the crude estimates (DENV, 98.3%; ZIKV, 99.6%) and similar to the values found in previous studies without correction, while the correct specificity (DENV, 66.7%; ZIKV, 77.1%) was lower than that observed in the systematic review study and in the accuracy study conducted between 2018 and 2019 with RT^8,24^.

According to our estimates, more than 80% of the population was exposed to DENV and approximately 42% to ZIKV. We found that correction of RT accuracy led to an important increase in the overall prevalence rates for DENV and ZIKV and yielded higher values than those observed in the previous study conducted without PRNT results, in which 1,001 volunteers positive for DENV and ZIKV were classified as flaviviruses^5^. Among the test parameters, the adjustment of sensitivity was the main factor responsible for the increase in seroprevalence, as it enabled the identification of a large number of previously exposed individuals.

Umemneku Chikere et al.^25^, mention the Begg and Greenes^20^ method as useful for correcting partial verification bias in which the results are easily interpretable. A recent study that showed partial verification bias used the same method and reported that specificity was higher than sensitivity, with minimal variation between the two tested methods^26^. The method assumes that some participants that were not verified by PRNT (gold standard) would show positive results if they were confirmed by PRNT. Thus, the estimates may increase slightly^18^. Previous studies^18,25,26^ applied the Begg and Greenes^20^ method and found that it presents reliable results for screening tests with binary results. The results in these studies were similar to the multiple imputation method, used to analyze incomplete databases, replacing missing data with multiple estimates^27^. Unlike imputation, the Begg and Greenes^20^ method deals directly with partial verification bias when selecting participants for the gold standard. However, we found no studies that used the Begg and Greenes^20^ method to assess diagnostic tests for arbovirus infections. This may be explained by the inherent complexity of the method itself, rather than by any characteristic of arboviruses^25^.

Our results differ from the values presented by the manufacturer of the DPP® RT for ZIKV and DENV, in which sensitivity is reported as close to 100% for both tests, but with high cross-reactivity^28^. The corrected RT sensitivity for ZIKV (91.5%) was higher than reported in the review study by Verotti et al.^8^, in which sensitivity varied from 10% to 87.5%, with lower values in areas with cocirculation of ZIKV and DENV, but there were proportionally few studies in endemic areas. In our study, the likelihood of having ZIKV increased fourfold, given a positive RT result. Our corrected RT sensitivity for DENV (81.0%) was also higher compared to other studies in areas with cocirculation of ZIKV and DENV, while our values for specificity were lower^6^. Our results showed better results for sensitivity for both tests, even in a scenario of flavivirus cocirculation. Our RT specificity values for DENV and ZIKV were lower than the values reported in the above-mentioned studies.

Rio de Janeiro is a city with flavivirus cocirculation, and the higher corrected RT sensitivity for both DENV and ZIKV is desirable for screening tests aiming to detect the disease in currently or previously infected individuals, reducing the rate of false-negative results. Such characteristic is important in the attempt to control ZIKV and DENV, underreported arboviruses with a high prevalence of asymptomatic infections. Adequate tests results in better flavivirus surveillance and development of appropriate actions and interventions.

We have previously shown lower estimations for the city of Rio without PRNT confirmation (48.6%, flavivirus; 28.9%, DENV; 3.2% ZIKV)^5^. By using the PRNT testing and the statistic corrections, we were able to better classify the flavivirus group, and the prevalences were much higher for both DENV (88.0%) and ZIKV (41.6%), consistent with previous prevalence results from a serological survey conducted among residents aged 5 to 65 years in Recife, Pernambuco state (DENV 88.7%; ZIKV 34.6%), between August 2018 and February 2019, the epicenter city of ZIKV-associated microcephaly in Brazil^29^.

Both Rio de Janeiro and the city of Recife are urban areas and endemic for DENV and ZIKV, with cyclical dengue epidemics and cocirculation of the arbovirus infections. The proportions of asymptomatic infections for DENV (80%) and ZIKV (50%) reported in areas with cocirculation^30^ were close to the corrected prevalence rates found in our study (DENV 88.0%; ZIKV 41.6%), thus emphasizing the importance of expanding the system for surveillance and monitoring of these arbovirus infections.

The prevalence of arbovirus infections was significantly higher in males and Black individuals, unlike the study by Braga et al.^29^, which found higher prevalence in females. We found that exposure to DENV increased considerably in the age groups of 19 years and older, with differences greater than 30% in some cases, potentially explained by longer exposure to DENV in older people compared to younger individuals^31^. Higher prevalence in males and increasing seroprevalence with age were reported in 41% of the DENV, ZIKV, and CHIKV seroprevalence studies worldwide analyzed by Fritzell et al.^31^

According to our estimates, more than half of the participants had been vaccinated for yellow fever, and the dengue and Zika prevalence rates were about 10% higher among unvaccinated individuals. The period from 2016 to 2018 witnessed the largest yellow fever outbreaks in Brazil, and the goal was to immunize 95% of the population in the recommended municipalities. YFV, DENV, and ZIKV belong to the flavivirus genus, and the serological tests may display cross-reactivity, potentially producing false-positive results. In this scenario, it is important to consider yellow fever vaccination status and the use of a differential diagnostic test such as PRNT, the gold standard for diagnostic confirmation, although yellow fever vaccination did not affect the estimates of ZIKV and DENV prevalence rates in our study. There are currently no reports of yellow fever cases in humans in Brazil^9^.

The correction of accuracy and DENV and ZIKV seroprevalence found in this study emphasizes that the true magnitude size of arbovirus infection burden in Brazil is still unknown. Our study found that Rio’s population had already been exposed to the three studied DENV serotypes, with DENV-3 as the most predominant. The results suggest the juxtaposition of DENV serotypes among participants and the overlapping of ZIKV and DENV, reported previously in MRJ^32^. DENV is the most prevalent arbovirus infection in the country, and up to the fourth epidemiological of 2024, the increase was greater than the peak observed in 2023^4^. A total of 201.1 dengue cases/100 thousand inhabitants were reported in the country, and the four DENV serotypes were identified, predominantly serotypes 1 and 2, with 62 confirmed deaths from the disease. Meanwhile, there was a 63% reduction in ZIKV cases compared to the same period in 2023^4^.

One of our study’s strengths is the correction of partial verification bias using PRNT as the gold standard and thus favoring our analyses’ reliability. PRNT is recommended for seroepidemiological surveys since it is more specific than Enzyme-Linked Immunoabsorbent Assay (ELISA) and discriminates cross-reactivity between flaviviruses^10^. However, our results should be interpreted with caution. Despite the actions taken in the study design to avoid selection and information bias, 769 out of 1,087 eligible participants for PRNT (70.7%) agreed to provide venous blood samples. Among those who refused, 62.0% (n = 197) were females, and 47.8% (n = 152) belonged to the 30 to 59-year age group. However, based on official reports, our sample seems to be representative of Rio, which counted with 53.0% of women and similar age distribution in 2018^33^. It was not possible to verify exposure to the DENV-4 serotype because of the difficulty in plaque formation in the PRNT. Two DENV-4 cases were identified in MRJ in the year 2018.

To minimize potential selection bias, census tracts from IBGE in MRJ were randomly selected, and within each tract, target households were also chosen at random. The selected areas were visited to update information from the 2010 national census, and each selected household was approached at least three times before being considered vacant, in which case it was replaced by another from a pre-defined list of residences. In the included households, all consenting residents were invited to participate, with additional visits scheduled on weekends to reach those absent during the week. Furthermore, potential selection and participation bias were corrected through the use of sampling weights and incorporation of the sampling design in the analyses.

Epidemiological surveys are generally expensive and difficult to perform^32^. Our study used two methods for the correction of partial verification bias in the serological test, based on a survey conducted previously with complex sampling, where negative RT results for the target arboviruses had not been confirmed by PRNT. Our study was pioneering in using the Begg and Greenes^20^ correction method in a scenario of cocirculation of the arbovirus infections. However, the formula of this method does not directly allow for the calculation of uncertainty intervals; therefore, an alternative approach was used to compute 95% confidence intervals for the corrected parameters; its application in our study allowed the correction of population prevalence rates of DENV and ZIKV. Calculations of 95%CI for estimated prevalence rates were performed using the survey statistical package^23^, which allows analyzing data from complex sampling and calculating the confidence intervals for the proportions. We highlight this study’s importance, since few seroepidemiological surveys are performed in Brazil.

## CONCLUSION

We concluded that the RTs for DENV and ZIKV used in our study displayed higher sensitivity than specificity, which was moderate, in a city with flavivirus cocirculation, and that the results are comparable to those of other studies in areas resembling and differing from the values reported by the manufacturer. The moderate specificity may be attributed to flavivirus cross-reactivity, underscoring that RTs can be used for screening target arbovirus infections. The prevalence of arbovirus infections reveals a scenario of even more serious exposure than reported in our previous study in Rio^5^. The implementation of the same methods may be useful in subsequent seroprevalence studies that present the limitation of partial verification bias, furnishing more robust results. Our results support the need for monitoring dengue and Zika cases and reinforce the importance of active surveillance to identify disease cases at an early stage and propose preventive measures before the wider circulation of the viruses in the population.
